# Combinatorial identification of DNA methylation patterns over age in the human brain

**DOI:** 10.1186/s12859-016-1259-3

**Published:** 2016-09-23

**Authors:** Behrooz Torabi Moghadam, Michal Dabrowski, Bozena Kaminska, Manfred G. Grabherr, Jan Komorowski

**Affiliations:** 1 Department of Cell and Molecular Biology, Computational and Systems Biology, Uppsala University, Uppsala, Sweden; 2 Laboratory of Bioinformatics, Neurobiology Center, Nencki Institute of Experimental Biology of Polish Academy of Sciences, Warsaw, Poland; 3 Laboratory of Molecular Neurobiology, Neurobiology Center, Nencki Institute of Experimental Biology of Polish Academy of Sciences, Warsaw, Poland; 4 Department of Medical Biochemistry and Microbiology/BILS, Genomics, Uppsala University, Uppsala, Sweden; 5 Institute of Computer Science, Polish Academy of Sciences, 01-248 Warszawa, Poland

**Keywords:** DNA methylation, Aging, Rule-based classification, Feature selection

## Abstract

**Background:**

DNA methylation plays a key role in developmental processes, which is reflected in changing methylation patterns at specific CpG sites over the lifetime of an individual. The underlying mechanisms are complex and possibly affect multiple genes or entire pathways.

**Results:**

We applied a multivariate approach to identify combinations of CpG sites that undergo modifications when transitioning between developmental stages. Monte Carlo feature selection produced a list of ranked and statistically significant CpG sites, while rule-based models allowed for identifying particular methylation changes in these sites.

Our rule-based classifier reports combinations of CpG sites, together with changes in their methylation status in the form of easy-to-read IF-THEN rules, which allows for identification of the genes associated with the underlying sites.

**Conclusion:**

We utilized machine learning and statistical methods to discretize decision class (age) values to get a general pattern of methylation changes over the lifespan. The CpG sites present in the significant rules were annotated to genes involved in brain formation, general development, as well as genes linked to cancer and Alzheimer’s disease.

**Electronic supplementary material:**

The online version of this article (doi:10.1186/s12859-016-1259-3) contains supplementary material, which is available to authorized users.

## Background

DNA methylation is an important epigenetic mechanism modifying mammalian genomes. It plays a major role in several biological phenomena, such as X chromosome inactivation, imprinting, regulation of gene expression, development, cell differentiation, and the onset and progression of multiple diseases. The predominant form is cytosine methylation (5mC) at CpG dinucleotides. These CpG sites are located nonrandomly in the genome, tending to occur within high density clusters of CpGs (islands). Around 70 % of all CpG dinucleotides are methylated [1–4]. CpG islands constitute around 60 % of human promoters and are predominantly unmethylated, while the sites in the remaining 40 % are often hypermethylated [5, 6]. DNA methylation patterns are known to be tissue specific [7], and it has been shown that one of the effects of DNA methylation is to contribute to transcriptional silencing, where proteins bind directly to methylated DNA and recruit co-repressor complexes [8], triggering the formation of repressive chromatin.

There are several studies that show that changes in DNA methylation are not confined to early development, but rather occur over the entire life span of an organism, resulting in distinct age-related methylation profiles [9–12]. While this global ‘epigenetic clock’ correlates strongly with chronological age and could thus be used to evaluate or exclude age-related factors in analyses of neurodevelopmental or neurodegenerative disorders, the specific genes or networks that depend on this clock for their regulation remain largely unknown. Since the changes in methylation patterns are complex and likely involve multiple genes in a combinatorial manner, in this study our goal was to explore how a non-linear multivariate machine learning approach, capable of analyzing multiple CpG sites simultaneously, would interpret the data without any *a priori* hypothesis such as the direction or trajectory in which the changes in methylation occur.

We thus applied a rule-based approach to a public methylation dataset profiled from the prefrontal cortex of the brain [13], for which we first examined changes across all age boundaries. After applying Monte Carlo Feature Selection [14] to rank the CpG sites by significance, we identified five distinct age groups, with marked transitions between them. We then used ROSETTA [15], which implements rough sets theory [16], to construct rule-based models based on the identified CpG loci.

## Methods

### Data preprocessing

The data set used in this work, Numata et al. [13], comprises DNA methylation data from 108 samples, taken from individuals ranging from fetal to 84 years old, designed to study the dependence of methylation on age and gender. Genomic DNA has been taken from dorsolateral prefrontal cortex. Illumina’s Infinium HumanMethylation27 BeadChip was used to profile the DNA methylation level at 27,578 CpG dinucleotides.

We removed sites from the dataset if they fulfilled one or more of the following conditions: (a) CpG sites fall on chromosome X; (b) Potentially nonspecific or polymorphic probes present on Infinium HumanMethylation27 BeadChip; or (c) CpG sites with standard deviation of beta values < 0.02 to remove uninformative sites. Beta values, which were measured from a population of cells and are therefore reported as average on a scale from 0 to 1, were discretized into: (a) *unmethylated* if the chip reports a beta value of 0.2 or lower; (b) *methylated* if the beta value is 0.8 or higher; and (c) *intermediate* if the beta value is between 0.2 and 0.8. Discretizing the beta values was motivated by Bibikova, Le, Barnes et al. [17], who divided the beta values into the three groups “methylated”, “hemimethylated”, and “unmethylated”, proposing the threshold values 0.2 and 0.8 based on the overall distribution of beta values (see Additional file 2: Figure S1).

### Decision tables and selecting significant CpG sites

We constructed decision tables as follows (see Table 1 for an example): each row represents a sample with the values of the characterized features of that sample in the columns. Here, features are the selected CpG sites with their methylation levels as measured by the chip. The last column holds the decision class the sample belongs to.

**Table 1:** A fragment of a decision table

SampleID/CpGID	CpG1	CpG2	…	CpGN	Class
Sample1	methylated	intermediate	…	unmethylated	youngerThan0
Sample2	intermediate	methylated	…	methylated	olderThan0
…	…	…	…	…	…
Sample108	unmethylated	methylated	…	unmethylated	youngerThan0

The rows show the samples and the columns show the value for each property (feature). The last column contains the decision (class) to predict

We constructed 61 separate binary decision tables (also referred as two-class decision tables) by iteratively dividing the samples into two groups: (1) younger than a given age, or (2) older than or equal to a given age. Since there were very few samples older than 60, we stopped the cuts at the age of 60. In order to find the CpG sites that significantly contribute to classifying the samples to the age classes, we applied Monte Carlo Feature Selection (MCFS) [14] to compute a normalized relative importance (RI-norm) score for each feature. MCFS compensates for any imbalanced number of objects in each class.

### Constructing classifiers

**Fig. 1:**
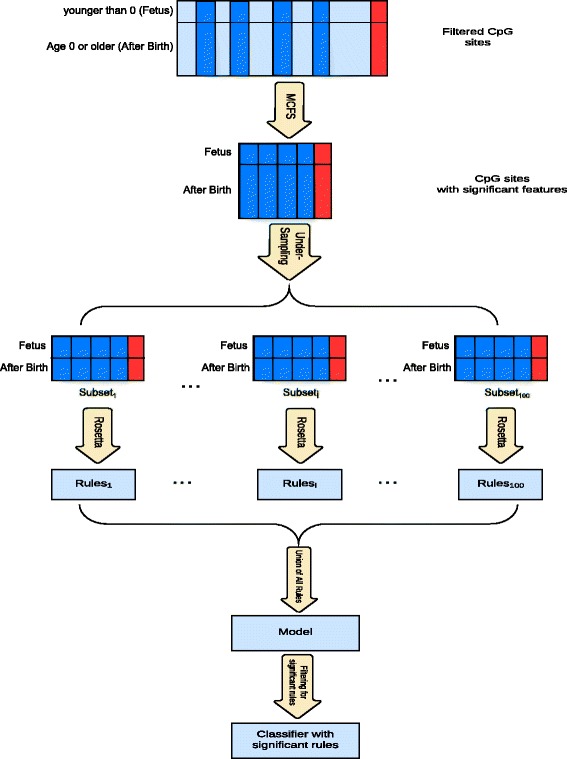
Summary of the analytical processes

Figure 1 illustrates a schematic overview of the method. The significant CpG sites for each age were extracted from the relevant decision table. Since the number of samples in different age classes was different, we ran a 100-fold under-sampling to avoid the bias of classification towards classes with more samples. In under-sampling, a new decision table is created, consisting of the samples of the smallest group, plus a randomly selected subset of samples (equal to the number of samples in the smallest group) from the other groups. This process was repeated 100 times.

We then created rule-based models using ROSETTA [15] for each under-sampled decision table, and combined the results into one model for each age class. ROSSETA implements rough set modeling [16, 18], the models of which are output in the form of human-readable IF-THEN rules, which describe the relation between the CpG sites and the decision. The rules are either c*onjunctive*, consisting of a conjunction of conditions, such as:

IF cg26158194=methylated AND cg12078929=intermediate

THEN ‘olderThan50’

Accuracy=0.95, support=21

Or *singleton*, having only one condition, such as:

IF cg10608341=unmethylated THEN ‘youngerThan0’

Accuracy = 1, support = 30

Each rule can be read as: if the condition(s) in the IF part is (are) satisfied for a sample, the model predicts it to be a member of the decision class (the THEN part of the rule). The reported accuracy shows the ratio of correctly classified objects to all classified objects to a specific class, based on the model confusion matrix. The support value shows the number of samples that satisfy the condition(s) (the IF part).

ROSETTA outputs a list of rules that are independent of each other, i.e. if multiple sites, or combinations thereof, act as “predictors”, it reports all of them. This, in turn, allows for interpreting the set of CpG sites used in the rules as being subject to changes in methylation at each specific age. To avoid over-fitting of the data, we computed the statistical significance for each rule using a hyper-geometric distribution and Bonferroni-correction, discarding rules with *p* > 0.05.

We used the web based tool Ciruvis [19] to identify interactions among rule conditions, i.e. rules that use the same CpG sites, ranked by *strength* - the sum of accuracies multiplied by support for all the rules in which it appears.

### Discretization into age groups

We computed the Jaccard distance between all two-class decision tables based on the number of overlaps (intersection) between the significant features obtained for each individual two-class decision table, i.e. given two Significant Features for Age sets SFA_i_ and SFA_j_ for decision classes i and j, the distance is computed as:

distance(SFA_i_, SFA_j_) = 1 – ((SFA_i_∩ SFA_j_)/(SFA_i_∪ SFA_j_))

### Annotation of sites and rules

We annotated the CpG sites using Annovar [20], allowing for identifying the genomic region in which a CpG site was located, using the tags exonic, intronic, UTR5, UTR3, intergenic, splicing (variant is within 2-bp of a splicing junction), and upstream (variant overlaps 1-kb region upstream of transcription start site). Functional annotation for the genes and the biological processes they are involved in was obtained from GeneCards (http://www.genecards.org).

## Results and discussion

### Informative CpG sites

**Fig. 2:**
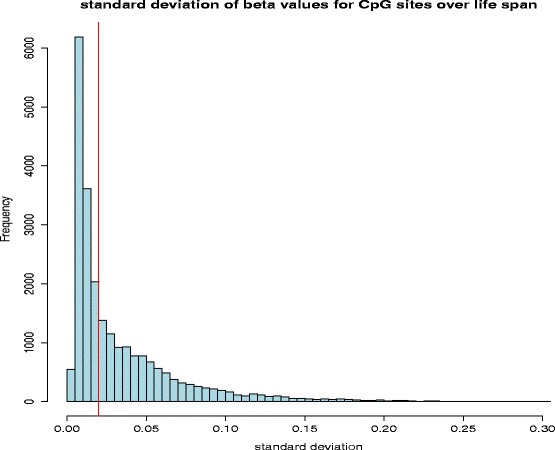
Histogram of standard deviation (SD) of beta values for all the CpG sites after discarding the sites located on the X chromosome or located on nonspecific or polymorphic probes

In the Numata et al. dataset [13], methylation levels measured by the Illumina Infinium HumanMethylation27 BeadChip are reported as *beta values*, i.e. the methylation averaged over the cell population that was sampled, at 27,600 CpG dinucleotide sites. Of these, we first removed 1086 sites located on chromosome X to avoid sites in pseudo-autosomal regions on chromosome Y in male participants. We next removed 770 polymorphic loci, and 2626 loci located on non-uniquely mapped probes (both according to the chip manifest file). For the remaining sites, we computed the standard deviation of beta values, and discarded sites that showed little variation in methylation throughout age, by applying a cutoff of 0.02 (Fig. 2), resulting in around 11,000 CpG sites.

### Discerning CpG sites over age

**Fig. 3:**
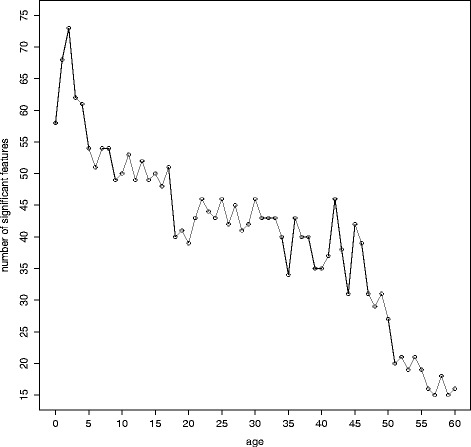
The number of significant CpG sites plotted for all two-class decision tables

For any given age, we next binned the samples into two categories, either below or above this age, and repeated the process 61 times for ages 0 to 60. We then applied Monte Carlo Feature Selection (MCFS) to the respective two-class decision table to determine the set of CpG sites that significantly (p < 0.05) contribute to the classification. The number of such sites decreased as age increases (Fig. 3), indicating that fewer sites are able to discern between samples at higher age and implying that changes in methylation are generally more pronounced at early ages and decrease with age.

We next examined the union of all significant CpG sites, 283 in total (Additional file 1), for functional categories. In this file, the coding is “0” for the sites not to be significant to discerning the age category, and “1” for the sites that are significant, marking genes also found by Numata et al. [13] in bold. Genes overlapping with these sites are involved in various developmental, proliferation and differentiation processes, but also in brain or neuron specific functions and diseases, as expected. A total of 51 CpG sites only contributed to the classification of age 0 (discerning fetus from after birth samples), consistent with the trend that was reported in the original study [13]. The specific genes associated with these include *ARTN, KMO, KCNA10, SNRK, SLC1A7, HAAO, FEV, CHRD, SPON2, HESX1, OTOF, ELAVL4* and *SULT1C2,* which are involved in brain and/or neuron specific processes, as well as *SLC1A7*, which is implicated in schizophrenia and other mental illnesses (http://www.genecards.org). The site annotated to a 5′UTR region of this gene showed an increase of methylation level from *intermediate* to *methylated* as age increases. A site that changed from unmethylated in fetus to intermediate in adulthood was located upstream of *FEV*, a gene implicated in the pathophysiology of psychiatric disorders such as depression, anxiety and eating disorders. Another site showing the same pattern is located in an intron of *SPON2*, which codes for a protein promoting adhesion and outgrowth of hippocampal embryonic neurons, as well as a site in the 5′ untranslated region of *HESX1*, which codes for a transcriptional repressor protein in the developing forebrain. We further identified genes involved in the regulation of processes such as cell migration, cell differentiation and cell progression, namely *S100A1*, *ARHGAP25* and *LIMS2*.

There were 13 sites exclusive to classifying age 1, which were associated with the genes *MSX1, MYL5, MPG, ACTN3, NAGS, DYDC1-DYDC2, SCN4B, HYDIN, TST, LFT, CTSZ, EMP3* and *NET1.* There were fewer sites involved in classifying older ages (50 years and above), located in genes such as *ANK1, HOXA9, TMEM61, ATP8A2, TEX264* and *TINAGL1*. Several CpG sites were involved in classification of a wide range of ages, some of which covered well-defined intervals. For example, the sites associated with *NPTX2* (upstream), *CHRNB4* (intron), *RAB42* (intron) and *ESRP2* (exon) were reported as significant for all ages between 19 and 60.

### Classification into age intervals

**Fig. 4:**
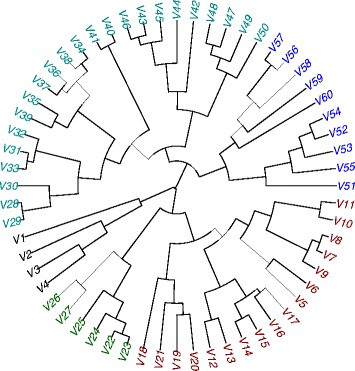
Hierarchical clustering of the significant CpG sites obtained from running MCFS on all two-class decision tables. The distance between every two significant CpG sets is based on the number of overlapping sites

Using the Jaccard distance as a measure for the similarity between the calculated significant CpG sites for each age above 0, we computed a full distance matrix and applied hierarchical clustering in R (hclust function with the ‘complete’ method, Fig. 4). There are three distinct groups comprising age 0 to 4, age 5 to 27, and age 28 to 60, with the latter exhibiting sub-groups within their respective clades. The overall topology also reflects the trend observed earlier, in that age groups become broader with increasing age, Following this clustering, we constructed a four-class decision table based the classes: ‘fetus’, ‘Age 0–4’, ‘Age 5–27’ and ‘Age28plus’. MCFS identified 71 significant CpG sites on this set, on which we trained a classifier using ROSETTA. The resulting model had an accuracy mean of 90 % (expected NULL model accuracy: 25 %), as measured by 10-fold cross-validation.

### Analysis of the rule models

**Fig. 5:**
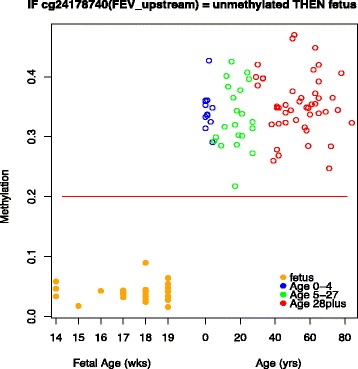
Plot of the beta values of all samples for cg24178740 annotated to the upstream of *FEV* that classifies *fetus* samples. The red line shows the margin between unmethylated and intermediate beta values. IF cg24178740 = unmethylated THEN ‘fetus’. Support is 30 and accuracy equals to 1 as all the objects classified as *fetus* turn out to be *fetus*

**Fig. 6:**
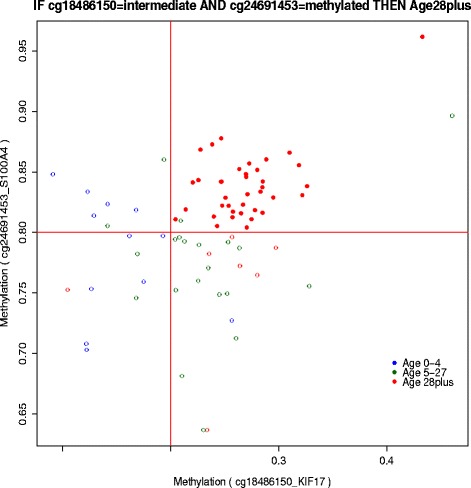
Plot of the beta values for two CpG sites appearing in the top significant rule for classifying *Age51plus*: IF cg27554782 = intermediate AND cg21053323 = intermediate THEN ‘Age51plus’. Accuracy equals to 0.92 and support equals to 25 for this rule. The red lines show the margin between unmethylated and intermediate beta values

The model consisted of a set of rules that classify samples to a decision class according to the values of the significant features (see Methods). For further analyzing these rules, we first filtered by significance (*p* < 0.05), accuracy (>75 %), and support (at least half the size of the support set). The top five most significant rules for each of the age classes are listed in Additional file 2. We tested the sites in each rule against a multivariate linear regression model, and highlighted the rules not significant in the regression in red. As expected, all singleton rules show significance in linear regression, whereas more than half of the conjunctive rules do not. Table 2 lists the confusion matrix, indicating that the classification power for fetus and age 0–4 is higher than the other groups. The fetus class comprised all of the singleton rules (see Methods), which is consistent with marked methylation level changes at birth, where single CpG sites are indicative for this transition (Fig. 5). By contrast, the majority of rules classifying the older age groups comprise were conjunctive, i.e. containing a combination of sites (Fig. 6).

**Table 2:** Confusion matrix for the rule-based classifier with four decision classes. The number of samples in each class is shown in parenthesis

Observed class	Classified as				
	Fetus	Age 0–4	Age 5–27	Age 28 plus	Accuracy per class
Fetus (30)	30	0	0	0	100 %
Age 0–4 (12)	0	12	0	0	100 %
Age 5–27 (21)	0	2	13	6	62 %
Age 28 plus (45)	0	1	1	43	95.5 %
Model accuracy					90 %

While the classifier for age 0–4 contained conjunctive rules with perfect classification power on the data set, the rule accuracy dropped for both the Age 5–27 and age 28+ classes, while the number of conjuncts increased, indicating that the changes in methylation become less dramatic and have thus less power to discern the samples by learning from their methylation patterns. When examining the direction in which methylation changes, we found that 81 % of the fetus rules showed a pattern of lower-to-higher methylation when transitioning into the next group. The associated CpG sites were linked to genes involved in cellular development, growth and proliferation, and neuronal related pathways and mechanisms. For example, the site (*cg00563926*)*,* located within the *TGFBR3* gene, whose decreased expression has been observed in various cancers [21], showed a methylation level increase from below 0.2 in fetus to 0.4 in adulthood. A similar pattern was observed for *cg22930187*, upstream of *ARTN*, which is a member of the glial cell-derived neurotrophic factor family of ligands, as was the site cg08965143 upstream of *TP53I3,* a gene induced by the tumor suppressor *P53*, which increased in methylation levels to 0.5 in adulthood (see Table 3 for top 10 singleton rules classifying fetus samples). Sites showing the reverse pattern were associated with *PLEK, PIK3C2B, RD3, KCNA10, ACAP3* and *GPR37L1* genes.

**Table 3:** List of 10 significant singleton rules classifying fetus samples

Rule
IF cg01561916(HAAO_upstream) = unmethylated THEN’fetus’
IF cg11618577(KRTCAP3_exonic) = unmethylated THEN’fetus’
IF cg18669381(ARHGEF19_UTR5) = unmethylated THEN’fetus’
IF cg04716261(ACTRT2_upstream) = unmethylated THEN’fetus’
IF cg16302441(POMC_upstream) = unmethylated THEN’fetus’
IF cg12467090(PIK3C2B_intronic) = methylated THEN’fetus’
IF cg19740375(SCN5A_intronic) = unmethylated THEN’fetus’
IF cg24178740(FEV_upstream) = unmethylated THEN’fetus’
IF cg20289949(HAAO_exonic) = unmethylated THEN’fetus’
IF cg07830847(KCNA10_exonic) = methylated THEN’fetus’

All of these rules are perfect rules with accuracy of 1 and support of 30 (all fetus samples)

The *cg00548268* site was annotated to the upstream region of *NPTX2.* This gene encodes neuronal pentraxin II (or neuronal activity-regulated pentraxin, Narp), which is involved in neuritic outgrowth, synapse remodeling and the aggregation of neurotransmitter receptors at synapses [21]. The *NPTX2* gene is reported to be hypermethylated in tumors, including brain tumors [22] and in this data showed an increasing pattern from 0.05 towards 0.2 from fetus towards age 5–27 class and up to 0.3 in the age 28+ class. The site annotated to *FBXO2* also appeared in the rules with a similar pattern. *FBXO2* is related to Alzheimer disease, as it regulates APP processing [24].

### Rules interactions

**Fig. 7:**
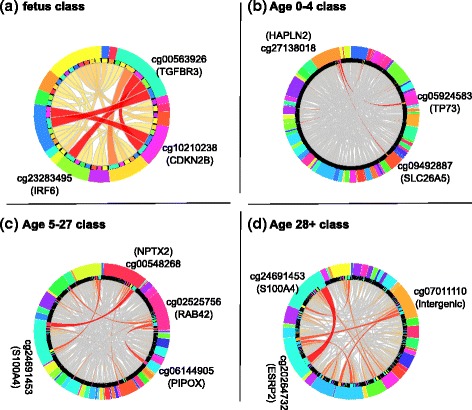
Top 2 strong interactions among the CpG sites for the rules, generated by Ciruvis, for fetus (**a**), Age 0–4 (**b**), Age 5–27 (**c**) and Age 28+ classes. The outer ring lists the CpG sites and the inner ring shows the sites connected (interacting) to each of them by an edge. The thicker and darker the edge, the stronger the interaction

We used Ciruvis [19] to investigate the combinations of the features in the rules. Figure 7 depicts the outcome for each of the decision classes. Two features (CpG sites) are connected inside the circle if they co-occur in multiple rules. The connections are shown as edges between the nodes. The width and color of the edges reflect the connection score (low = yellow and thin, high = red and thick). Table 4 lists the two strongest interactions for all classes.

**Table 4:** Top two strongest interactions for different decision classes

Interactions	Class
cg00563926(TGFBR3) = unmethylated AND cg23283495(IRF6) = unmethylated	Fetus
cg00563926(TGFBR3) = unmethylated AND cg10210238(CDKN2B) = unmethylated	Fetus
cg05924583(TP73) = unmethylated AND cg27138018(HAPLN2) = intermediate	Age 0–4
cg27138018(HAPLN2) = intermediate AND cg09492887(SLC26A5) = unmethylated	Age 0–4
cg24691453(S100A4) = intermediate AND cg00548268(NPTX2) = unmethylated	Age 5–27
cg02525756(RAB42) = unmethylated AND cg06144905(PIPOX) = intermediate	Age 5–27
cg24691453(S100A4) = methylated AND cg20264732(ESRP2) = intermediate	Age 28plus
cg07011110(intergenic: LOC285819, BTN1A1) = methylated AND cg20264732(ESRP2) = intermediate	Age 28plus

Some of these interactions indicate coordinated age-related changes in methylation of genes involved in the same biological function or process. For example, gene *TGFBR3* encodes TGF beta-receptor III (also known as betaglycan) that serves as a co-receptor for other types of TGF beta-receptors. Conversely, ectodomain shedding of *TGFBR3* produces soluble *TGFBR3*, which inhibits TGF beta signaling [24]. In either case, changes in the expression of TGFBR3 are likely to affect the signaling through the TGF beta pathway. The other gene listed as interacting with *TGFBR3*, namely *CDKN2B*, encodes cyclin-dependent kinase inhibitor 2B, which is a potent inhibitor of the cell cycle. In epithelial cells, *CDKN2B* is known to be induced by TGF beta [25]. The link between *TGFBR3*, *CDKN2* and the cell cycle is likely relevant to the aging brain, as TGF beta stimulates proliferation of microglia [26], brain resident macrophages, which play important role during aging.

Another strong interaction lists gene *TP73* together with *HAPLN2*. Gene *TP73* encodes the tumor protein 73, a member of the p53 family of transcription factors, with a role in neuronal differentiation and hippocampal development [27]. Its deficiency results in impaired self-renewal and premature differentiation of mouse neuronal progenitors [28]. *TP73* is also a major survival factor for postmitotic neurons [30]. Gene *HAPLN2* encodes brain-specific hyaluronyan and proteoglycan link protein 2. This protein in the cerebellar cortex is produced by neurons and localizes in the perineural net [30]. *HAPLN2* also localizes at the nodes of Ranvier in the myelinated regions of the developing central nervous system [31], where it plays a role in the formation of the cation diffusion barrier, important for the conduction velocity [32]. The processes of neuronal differentiation and formation of the myelin sheet are related, which may explain why the genes interact.

Several of the genes appearing in other interacting rules have been previously individually associated with aging. The methylation of *NPTX2* was found to correlate with the chronological age, with older individuals having enhanced methylation [34]. The gene *ESRP2*, appearing in two rules for the ‘Age 28plus’ class, controls adult-specific splicing program in mouse hepatocytes [34]. The gene *S100A4*, which encodes the S100 calcium binding protein A4 and is involved in the regulation of neuritogenesis and neuronal survival [35], was previously identified to be regulated in a telomerase-dependent way [36].

## Conclusions

We applied machine learning techniques to identify genes that contain CpG sites that change in methylation levels or patterns at particular boundaries over life span. Our approach does not aim at developing a classifier of age itself - there are other methods that can predict age using the methylation status [12–37] - but rather it introduces a method to explore which combinations of CpG sites and associated genes contribute to changing patterns at the given boundaries and methylation levels as the age change. Not surprisingly, changes in methylation were most pronounced at birth, as has been reported earlier [13], resulting in comprehensive singleton rules using our approach. Conjunctive rules, i.e. rules that associated more than one CpG site and its methylation status with an age class, showed the combinatorial role that methylation may play in more interconnected ways. Our results reported that the patterns of methylation changes in a healthy individual’s brain were highly complex and interdependent. While we confirmed 33 genes related to aging or involved in diseases, notably cancer, Alzheimer’s disease, and autism, that have been reported in the original study [13], we identified a number of additional genes, many of which are linked to developmental and/or nervous systems specific function. In conclusion, we expect that future studies will adopt our, or a similar machine learning approach to test these hypotheses by utilizing a multivariate analysis to compile a network of candidate CpG sites and associated genes.

## Additional files

Additional file 1: The union of significant CpG sites (annotated) for all age cuts, showing 1 if a specific site is significant for a specific age. (XLSX 67 kb) Additional file 2: Figure S1. Histogram of beta values after filtering for standard deviations across all samples. **Figure S2.** Histogram of the samples’ ages. **Table S3.** Top 5 rules classifying the ‘fetus’ class. **Table S4.** Top 5 rules classifying the ‘Age 0To4’ class. **Table S5.** Top 5 rules classifying the ‘Age 5To27’ class. **Table S6.** Top 5 rules classifying the ‘Age 28plus’ class. (DOCX 82 kb)

## Abbreviation

MCFS: Monte Carlo feature selection
