# Supplementary material for: Combinatorial identification of DNA methylation patterns over age in the human brain
Source: BMC Bioinformatics. 2016 Sep 23;17:393. doi: 10.1186/s12859-016-1259-3 (PMC5034667; doi:10.1186/s12859-016-1259-3)
Supplement: Additional file 2: Figure S1. — Histogram of beta values after filtering for standard deviations across all samples. Figure S2. Histogram of the samples’ ages. Table S3. Top 5 rules classifying the ‘fetus’ class. Table S4. Top 5 rules classifying the ‘Age 0To4’ class. Table S5. Top 5 rules classifying the ‘Age 5To27’ class. Table S6. Top 5 rules classifying the ‘Age 28plus’ class. (DOCX 82 kb) [file 12859_2016_1259_MOESM2_ESM.docx]

Supplementary Figure 1 - Histogram of beta values after filtering for standard deviations across all samples.

Supplementary Figure 2 – Histogram of the samples’ ages.

Supplementary Table 1 - Top 5 rules classifying the 'fetus' class.

| If part | Accuracy | Support |
| --- | --- | --- |
| cg07830847(KCNA10_exonic)=methylated | 1 | 30 |
| cg00658007(RD3_UTR5)=methylated | 1 | 30 |
| cg01561916(HAAO_upstream)=unmethylated | 1 | 30 |
| cg24178740(FEV_upstream)=unmethylated | 1 | 30 |
| cg04716261(ACTRT2_upstream)=unmethylated | 1 | 30 |

Supplementary Table 2 - top 5 rules classifying the 'Age 0To4' class.

| If part | Accuracy | Support |
| --- | --- | --- |
| cg08970446(SLC1A7_UTR5)=methylated AND cg06144905(PIPOX_upstream)=unmethylated | 1 | 12 |
| cg26227005(ELAVL4_intronic)=intermediate AND cg06144905(PIPOX_upstream)=unmethylated * | 1 | 12 |
| cg08970446(SLC1A7_UTR5)=methylated AND cg23283495(IRF6_upstream)=unmethylated AND cg27554782(CHRNB4_intronic)=unmethylated ** | 1 | 11 |
| cg08970446(SLC1A7_UTR5)=methylated AND cg27554782(CHRNB4_intronic)=unmethylated AND cg19224278(ALDH1A3_upstream)=unmethylated | 1 | 11 |
| cg08970446(SLC1A7_UTR5)=methylated AND cg04428453(ACSM5_upstream)=methylated | 1 | 10 |

* The condition ‘cg06144905(PIPOX_upstream)=unmethylated’ appears in combination with other CpG sites all with an ‘intermediate’ status, annotated to genes such as: ANGPTL1, RD3, LIMS2, DISP1, RPE65, HAPLN2, SULT1C2, HAAO, KRTCAP3, FEV and SCN5A, with the same accuracy and support.

** The conjunctive rules in red are the ones that could not be identified using a multiple linear regression model.

Supplementary Table 3 - top 5 rules classifying the 'Age 5To27' class.

| If part | Accuracy | Support |
| --- | --- | --- |
| cg02525756(RAB42_intronic)=unmethylated AND cg00548268(NPTX2_upstream)=unmethylated AND cg26538442(CES3_upstream)=methylated | 1 | 12 |
| cg02525756(RAB42_intronic)=unmethylated AND cg25462291(HEYL_intronic)=intermediate AND cg24691453(S100A4_upstream)=intermediate * | 1 | 11 |
| cg02525756(RAB42_intronic)=unmethylated AND cg25462291(HEYL_intronic)=intermediate AND cg00548268(NPTX2_upstream)=unmethylated | 0.93 | 14 |
| cg02525756(RAB42_intronic)=unmethylated AND cg25462291(HEYL_intronic)=intermediate AND cg20264732(ESRP2_exonic)=unmethylated | 0.93 | 14 |
| cg02525756(RAB42_intronic)=unmethylated AND cg00548268(NPTX2_upstream)=unmethylated AND cg19224278(ALDH1A3_upstream)=intermediate | 0.92 | 13 |

* The conjunctive rules in red are the ones that could not be identified using a multiple linear regression model.

Supplementary Table 4 - top 5 rules classifying the 'Age 28plus' class.

| If part | Accuracy | Support |
| --- | --- | --- |
| cg02525756(RAB42_intronic)= intermediate AND cg24691453(S100A4_upstream)=methylated * | 1 | 34 |
| cg24691453(S100A4_upstream)=methylated AND cg00548268(NPTX2_upstream)=intermediate | 1 | 31 |
| cg24691453(S100A4_upstream)=methylated AND cg27337148(CAMK1G_upstream)= intermediate AND cg20264732(ESRP2_exonic)= intermediate ** | 1 | 34 |
| cg18486150(KIF17_upstream)=intermediate AND cg24691453(S100A4_upstream)=methylated | 0.95 | 40 |
| cg24691453(S100A4_upstream)=methylated AND cg21104946(WNT7B_upstream)= intermediate | 0.95 | 40 |

* The conjunctive rules in red are the ones that could not be identified using a multiple linear regression model.

** The conjunct of “cg24691453(S100A4_upstream)=methylated AND cg20264732(ESRP2_exonic) = intermediate” appear in the rules in combination with other CpG sites annotated to different genes with the same accuracy and support.
